# Oral Contraceptive Steroids Promote Papillary Thyroid Cancer Metastasis by Targeting Angiogenesis and Epithelial-Mesenchymal Transition

**DOI:** 10.22088/IJMCM.BUMS.10.3.218

**Published:** 2022-01-10

**Authors:** Mohammad Hossein Dehghan, Mohammad Reza Ashrafi, Mehdi Hedayati, Setareh Shivaee, Sadegh Rajabi

**Affiliations:** 1 *Department of Biochemistry, School of Medicine, Alborz University of Medical Sciences, Karaj, Iran.*; 2 *Department of Biochemistry, Afzalipoor Faculty of Medicine, Kerman University of Medical Sciences, Kerman, Iran.*; 3 *Cellular and Molecular Endocrine Research Center, Research Institute for Endocrine Sciences, Shahid Beheshti University of Medical Sciences, Tehran, Iran.*; 4 *Traditional Medicine and Materia Medica Research Center (TMRC), Shahid Beheshti University of Medical Sciences, Tehran, Iran.*

**Keywords:** Papillary thyroid cancer, metastasis, migration, angiogenesis, epithelial-mesenchymal transition

## Abstract

Thyroid cancer is the most prevalent type of endocrine malignancy with the highest incidence rate among women under 45 years old. Ethinylestradiol (EE) and levonorgestrel (LNG) are two steroid components of low-dose oral contraceptives used all over the world. In this study, we aimed to examine the possible effects of the combination of these two steroids on metastasis and angiogenic factors in BCPAP papillary thyroid cancer (PTC) cell line. After treatment of BCPAP cells with the combination of 20 nM EE and 90 nM LNG, mRNA expression levels of long noncoding RNAs *HOTAIR* and *MALAT1*, angiogenic and antiangiogenic gene markers *VEGFA* and *THBS1*, and epithelial-mesenchymal transition (EMT) biomarkers *CDH1*, *CDH2*, *FN1*, and *VIM* were analyzed by real-time PCR. Additionally, the protein expression of VEGFA was semiquantified by Western blotting. Results showed that the combination of LNG and EE significantly elevated the level of VEGFA protein and mRNA expression of *VEGFA, MALAT1, HOTAIR, CDH2, FN1,* and *VIM* genes while decreased *CDH1* gene expression but had no marked effect on the expression of *THBS1* gene in comparison with the control group. Also, our results suggest that LNG and EE may increase the metastatic and migratory properties of BCPAP cells via modulating angiogenic and EMT biomarkers. These data may highlight the potential of exogenous steroids in the advancement of PTC tumors.

Thyroid cancer (TC) is the most common type of endocrine malignancies and its incidence is growing worldwide. TC incidence is the highest among women aged under 45 years old (1). It has been revealed that TC is the second most incident cancer type in pregnant women after breast cancer (2). Papillary thyroid carcinoma (PTC) is the most common form of thyroid carcinoma comprising around 70% of all cases (3). PTC often develops at younger ages and has 2.9 fold higher incidence in women than men (4). Higher incidence of PTC in younger women could propose a role for sex hormones especially estrogen and progestins (whether endogenous or exogenous) in the development and progression of this type of cancer (5). Low dose (LD) contraceptives comprising levonorgestrel (LNG) and ethinylestradiol (EE) are widely used as birth control tablets in Iran and most European countries (6, 7). Angiogenesis is a process by which tumor cells achieve their vast need for oxygen and nutrients supply. Vascular endothelial growth factor (VEGF) is the main protein responsible for the angiogenesis process, and its expression is elevated during this process in tumors. Trombospondin1 (*THBS1*) encodes a protein involved in reversing the above-mentioned process and is an anti-angiogenic factor (8). Long non-coding RNAs (lncRNAs) are more than 200 nucleotides long RNAs that have implications in the regular development and tumorigenesis process (9). Metastasis-associated lung adenocarcinoma transcript1 *(MALAT1*) encodes the lncRNA transcript that regulates the VEGF-mediated angiogenesis process (9). HOX transcript antisense RNA (*HOTAIR*) encodes lncRNA that regulates many cellular processes like proliferation, migration, and also angiogenesis process through regulating VEGF (10, 11). Epithelial-mesenchymal transition (EMT) is a process with a critical role in the migration and metastasis of cancer cells (12). Fibronectin1 (*FN1*), as a component of the extracellular matrix (ECM), plays a pivotal in the EMT process (13). Vimentin (VIM), as a component of the cytoskeleton, could be suggested as a biomarker for the EMT process (14), and cadherin 1 (CDH1) is a key protein involved in the EMT process due to its functions in cell-cell interactions, and reduced expression of *CDH1* gene is associated with EMT initiation (15). Cadherin 2 (CDH2) is another calcium-dependent adhesion molecule that is elevated during EMT causing the interruption of cell-cell interactions (16). Our previous work demonstrated that LNG in combination with EE could induce proliferation and invasion of BCPAP cells while inhibited apoptosis of these cancer cells (1). In this study, we aimed to study the effects of combined LNG and EE (as LD-combined oral contraceptives, OCPs) on angiogenesis and EMT processes in PTC cell line, BCPAP.

## Materials and methods

**Cell culture**

BCPAP, as a PTC cell line, was provided from National Cell Bank of Pasteur Institute (Tehran, Iran). Roswell Park Memorial Institute (RPMI) 1640 medium was used as the cell culture medium with the supplementation of 10% fetal bovine serum (FBS, from Gibco, Germany) and 1% penicillin/streptomycin (Biosera, England) (17). By achieving 60-70% cell confluency, the medium was replaced by a phenol red-free RPMI 1640 medium with 10 % charcoal-stripped FBS (Sigma Chemical, St. Louis, MO) and incubated for 24 h (18). Subsequently, the cultured cells were treated with 20 nM EE + 90 nM LNG (Aburaihan Company, Tehran, Iran).

**Quantitative real-time polymerase chain rea-ction (QRTPCR)**

To quantify the expression of our tested genes using QRTPCR method, BCPAP cells were seeded in 6-well culture plates at a density of 25×10^4^ cells/well. As described in our previous work, the concentrations of EE and LNG (20 nM EE + 90 nM LNG) were calculated based on their doses in LD-OCP tablets (1). To find an optimum treatment time period to obtain the best results, the cells were treated with the mentioned doses of the drugs for 24, 48, and 72 h. Consequently, 48 h treatment was found as the most optimum time for treating the cells. Control cells were treated with the normal medium for 48 h only. Then, total RNA was extracted by RNeasy Mini, RNA isolation kit (Qiagen, Germany) according to the manufacturer’s instructions. By using a Nanodrop 2000c spectrophotometer (Thermo Scientific, USA), the concentration of extracted RNA was calculated. Afterwards, cDNA was synthesized by cDNA Synthesis Kit (Bio FACT, Daejeon, South Korea). Alterations in the mRNA expressions of *VEGFA*, *THBS1, MALAT1, HOTAIR, CDH1, CDH2, FN1,* and *VIM* genes and beta-2-microglobulin (β2M), as internal control, were measured by quantitative real time PCR (qRT-PCR) in a rotor gene 6000 Corbett (Corbett Research, Sydney, Australia) detection system SYBR GREEN^®^ (nonspecific DNA-binding factors) (19). All primer sequences utilized in this present investigation are presented in Table 1. The fold changes and normalization of the above-mentioned genes were calculated by using LinReg (LinReg version 2012.1, Netherlands) and Relative Expression Software Tool (REST) softwares (Qiagen, Germany), respectively (1, 20).

**Western blotting**

For analyzing the expression of VEGFA protein, BCPAP cells were seeded in 6-well culture plates at a density of 25×10^4^ cells/well. As mentioned before, the cells in the treatment group were exposed to 20 nM EE and 90 nM LNG, but the control cells were just treated with the normal medium for 48 h. Next, the treated cells were collected using trypsin solution (1%), washed with PBS, and lysed by the radioimmunoprecipitation assay (RIPA) buffer 70 mM Tris-HCl (pH 7.4), 100 mM NaCl, 0.5% sodium deoxycholate, 0.1% SDS, 1.5 μM pefabloc), and then incubated on ice for 30 min while shaking. The cell lysates were then centrifuged at 15000 rpm for 20 min at 4 °C to collect the supernatants. The protein content was then measured by Bradford method and 40 μg of protein was separated by SDS- PAGE. Subsequently, they were transferred to the nitrocellulose membranes. After blocking by 5% skimmed milk in TBST (200 mM Tris–HCl, pH 7.4, 100 mM NaCl, and 0.05% Tween-20) for 3 h on a shaker at room temperature, the membranes were incubated with primary antibodies including VEGFA and beta-actin (Santa Cruz Biotechnology, Santa Cruz, CA) overnight on a shaker at 4 °Cin TBST. Then, the membranes were washed three times with TBST and incubated with the corres-ponding horseradish peroxidase (HRP)-conjugated secondary antibody for one hour at room temperature in TBST comprising 1% milk. 3, 3′-diaminobenzidine (DAB) solution (Sigma Chemical, St. Louis, MO) and 0.3% hydrogen peroxide (Merck, Germany) were used as substrates for developing membranes. Finally, the reaction was stopped by washing the blot with water and their images were semi-quantified using Image J software (21, 22).

**Table 1:** Primer sequences used in this study

**Gene name**	**Forward primer (5’ to 3’)**	**Reverse primer (5’ to 3’)**
***VEGFA***	GAGCAAGACAAGAAAATCCC	CCTCGGCTTGTCACATCTG
***MALAT1***	GCTCTGTGGTGTGGGATTGA	CTCGGGCGAGGCGTATTTAT
***HOTAIR***	AGACGAAGGTGAAAGCGAACC	CCCTCTGCCACGTTTGTTCC
***THBS1***	CCCTTGTGCTCAGAGTGGAT	GCCAGTAGAGAACAAATAAGCATGG
***CDH1***	GGGGTCTGTCATGGAAGGTG	CGACGTTAGCCTCGTTCTCA
***CDH2***	GCGTCTGTAGAGGCTTCTGG	GCCACTTGCCACTTTTCCTG
***FN1***	ACAAGCATGTCTCTCTGCCAA	TCAGGAAACTCCCAGGGTGA
***VIM***	TCCGCACATTCGAGCAAAGA	ATTCAAGTCTCAGCGGGCTC
***β2M***	TGTCTTTCAGCAAGGACTGGT	TGCTTACATGTCTCGATCCCAC

**Statistical analysis**

All data are expressed as mean ± SD, and were repeated at least three times. Statistical significance and differences between groups were analyzed using Student’s t-test, and Mann–Whitney U test. P < 0.05 was considered a significant value. The data were analyzed using GraphPad Prism 7 software.

## Results

**Effect of the combination of LNG and EE on angiogenesis-related factors expression**

**Fig.1:**
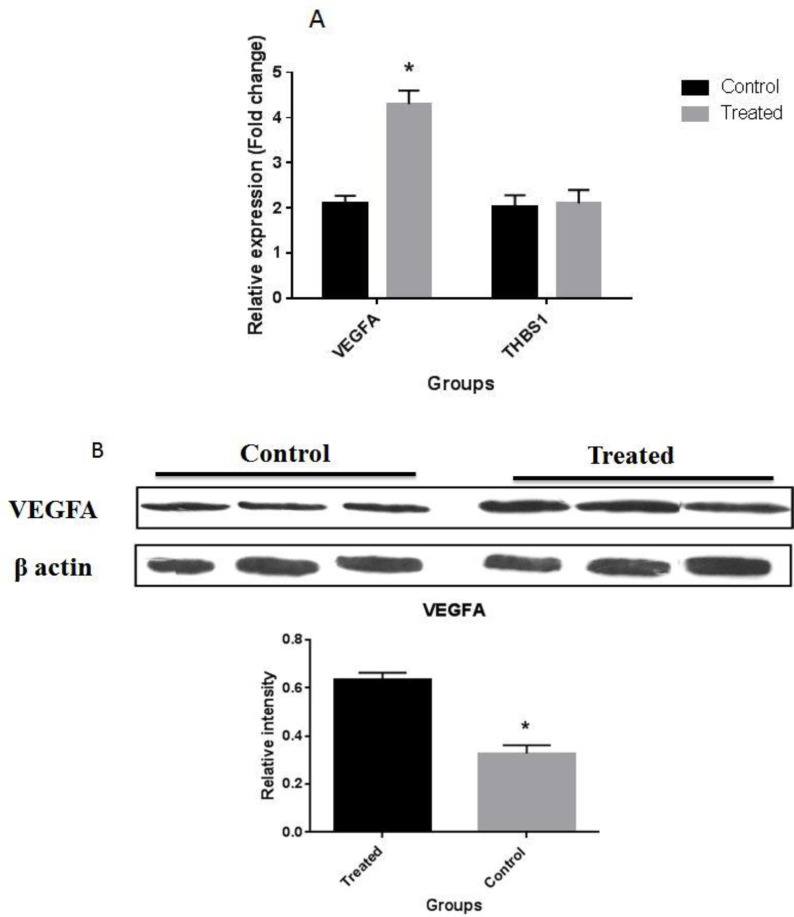
**Effect of the combination of LNG and EE on** ***VEGF*** **and** ***THSB*** **expression.** A the expression of *VEGFA* in treated group was increased compared to the untreated group while mRNA expression of *THBS1* did not change significantly between the two groups; B: the protein level of VEGFA was significantly elevated in treated grop compared t the untreated control group.. As illustrated in the figure, treated cells had higher expression of VEGFA protein in comparison with controls. All data are presented as mean ± SD. *P < 0.05 was considered a statistically significant level

As depicted in Figures 1A and B, treatment of BCPAP cells with the combination of 20 nM EE and 90 nM LNG significantly amplified the expre-ssion level of *VEGFA* gene in comparison with unt- reated control cells. Western blotting results showed that this treatment also significantly enhanced the VEGFA protein levels in comparison with the control cells. Further results indicated that these two steroids had no considerable effect on the expression of *THBS1* gene compared with the control.

**Effect of EE and LNG treatment on** ***MALAT1*** **and** ***HOTAIR*** **expression**

**Fig.2:**
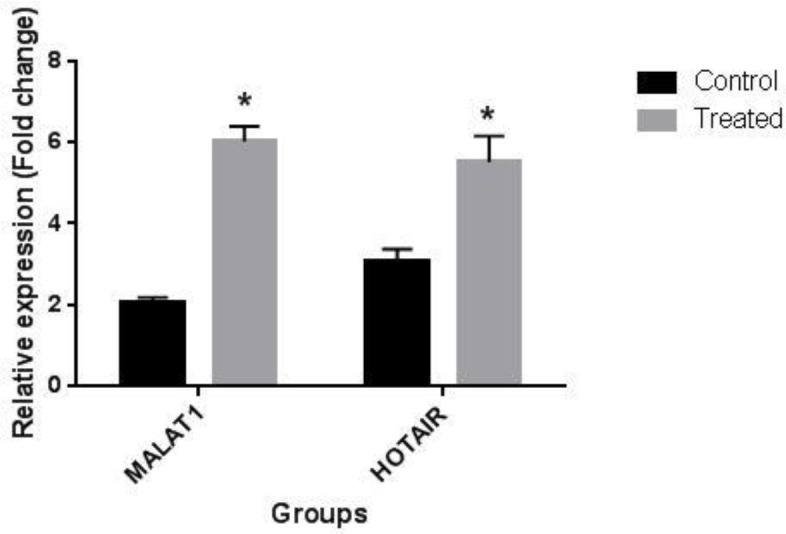
**Effect of EE and LNG treatment on** ***MALAT1*** **and** ***HOTAIR*** **expression.** Elevated expression of *MALAT1* and *HOTAIR* genes was observed in treated group in comparison with the control group. The data are representative of the mean ± SD of three independent experiments. *P<0.05 was considered a statistically significant level

To evaluate the possible role of an epigenetic mechanism on the angiogenic effect of LNG and EE on BCPAP cells, the expression levels of two key angiogenesis-related lncRNAs,* MALAT1 *and* HOTAIR* were measured. BCPAP cells were treated with 20 nM EE and 90 nM LNG in the treatment group and with the normal medium in the control group. Then, the expression levels of the mentioned lncRNAs were measured. As shown in Figure 2, mRNA expression of both* MALAT1 *and* HOTAIR* increased in EE and LNG treated group in comparison with the untreated control group. 

**Effect of the combination of LNG and EE on EMT gene markers expression**

**Fig.3:**
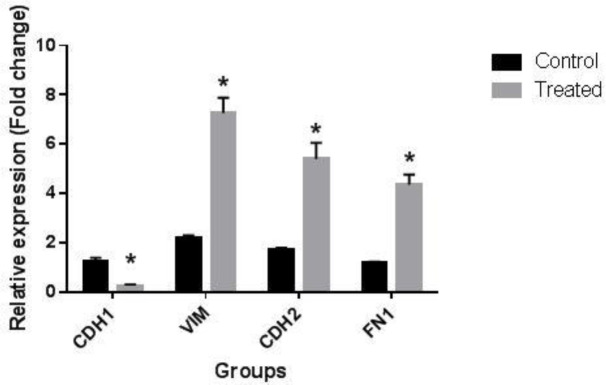
**Expression of EMT gene markers in the presence of LNG and EE.** The combination of LNG and EE treatment significantly reduced the expression level of *CDH1* gene in treated group compared to the control group while expressions of *CDH2*, *FN1*, and *VIM* genes were meaningfully increased in EE and LNG treated group compared to the control group. All data are presented as mean ± SD. *P < 0.05 was considered a statistically significant level

The expression levels of four key genes involved in the process of EMT were further measured to evaluate the implication of this process in the metastatic properties of LNG and EE in BCPAP cells. Figure 3 shows the effect of the treatment of BCPAP cells with the combination of LNG and EE on the expression of *CDH1, CDH2, FN1,* and *VIM* genes. The expression level of *CDH1* gene was lower in EE and LNG treated group in comparison with the control group while mRNA levels of *CDH2*, *FN1,* and *VIM* were higher in the treated group when compared with the control group.

## Discussion

Some previous evidence reveals that estrogen receptor alpha (ERα) is expressed at high levels in women with TC who consume OCPs and in well-differentiated TC patients (17). Furthermore, it has been documented that EE and LNG could upregulate *ERα *gene (23, 24). Interestingly, *ERα *can induce the production of VEGF in a variety of cancer cells (25). These facts are in line with our results which showed that treatment of PTC cells with EE and LNG induced the production of VEGFA. This indicates the potential role of these two exogenous steroids in triggering angiogenesis of PTCs, the process that is essential for tumor metastasis and advancement. 

Tao *et al*. showed that estrogens can up regulate HOTAIR (26). Therefore, according to the present data, we may imply that LNG and EE can induce the expression of *VEGF* by upregulating *HOTAIR *(11). Unchanged expression of *THBS1* in the present study may indicate that VEGFA-dependent pathway is the major signaling involved in the angiogenic activity of EE and LNG in PTC tumors.

*MALAT1 *RNA could also induce the EMT process in different cancer cells including lung, breast, and colon (27-29). In the present study, we found that treating BCPAP cells with the combination of LNG and EE remarkably changed the expression of EMT-associated gene markers, and this may contribute to the trigger of EMT in these cancer cells. Upregulation of *MALAT1* in the present investigation may be a potential epigenetic mechanism to induce the EMT process. Also, it has been shown that single nucleotide polymorphism (SNP) in *MALAT1* gene could cause susceptibility to PTC in Chinese population, and this may confirm the vital role of this gene in different aspects of initiation and progression of PTC (30). *MALAT1 *is capable of inducing *VIM *gene expression in hepatocellular carcinoma leading to the increase in migration and invasion of these cells (31). Thus, overexpression of *VIM* in our treated group could be due to an increased level of *MALAT1* gene expression (31). In our previous study, we carried out wound healing assay to indicate migratory effects of LNG and EE treatment on BCPAP cells (1). In the EMT process, expression levels of *CDH2* and *FN1 *are elevated, and these data were also observed in our study following the treatment of the cells with the mentioned steroids (32).

Increased expression of fibronectin along with α_5_β_1 _integrin enhances the angiogenesis process in human tumors in a VEGF-independent pathway (33). In our study, both *VEGFA* and *FN1* levels increased which could be indicative of an enhanced angiogenesis process, but it is not clear whether *MALAT1* and *HOTAIR* –induced *VEGFA* caused an elevated *FN1 *expression or another mechanism was involved. On the other hand, it has been documented that the EMT process itself has the potential to induce angiogenesis process via VEGF upregulation in breast cancer cells (34). Regarding the positive alterations of EMT biomarkers in our study in favor of the EMT-triggered metastasis induction, it seems that the upregulation of VEGFA protein in our treated group could also be interconnected to the EMT process (34). *CDH2* gene upregulation in cancer cells also induces angiogenesis via modulating VEGF. MAPK/ERK signaling pathway has been unraveled to play a critical role in this process (35). Moreover, it has been revealed that *MALAT1* could induce MAPK/ERK pathway in some cancer cells (36). This may link *MALAT1* and *CDH2* in an important signaling pathway, and suggests that *MALAT1* may act upstream of *CDH2*. Vimentin has the potential to induce the angiogenesis process, and since *MALAT1* is able to stimulate *VIM* gene expression, we may conclude that *MALAT1* acts as one of the major regulators of angiogenesis process by affecting *VIM* gene expression or its protein product (31, 37).

Estrogens like estradiol induce the expression of *HOTAIR *gene via ERα response element and cause the progression of sex hormone-dependent cancers like breast carcinoma (38). ERα is also able to activate MAPKs in different estrogen-dependent human cancer cells including uterine leiomyoma, endometrial, and breast (39-41). This ability of ERα can stimulate the angiogenesis process in a variety of cancers (35). This fact along with the regulation of* HOTAIR *by estrogens via miR-148a could emphasize the importance of *HOTAIR* upregulation in the induction of angiogenesis and progression of estrogen-dependent cancers such as thyroid and breast cancers (5, 26). Consistent with our results, Hernandez-Vega, *et al*. uncovered that estradiol has the potential to induce EMT by increasing the expression of *VIM* and *CDH2* genes and enhancing the migration and invasion of glioblastoma cells (42). Also, it has been reported that ERα is able to induce fibronectin and inhibit *CDH1 *expression in squamous cell carcinoma to facilitate the EMT process (43). Furthermore, estrogen-activated ERα has the potential to repress *CDH1 *gene by binding to the estrogen response element in the promoter region of this gene in breast cancer cells (44). These results are in line with our findings which showed that EE and LNG treatment caused *FN1* upregulation and *CDH1* downregulation. Overall, EE and LNG seem to exert their metastasis-inducing effects on BCPAP cells by two possible mechanisms; first by direct effect on the regulation of both VEGFA protein and EMT markers, and second by indirect effect on *MALAT1* and *HOTAIR*, which may alternatively modulate VEGFA and EMT markers.

Considering our data, estrogen and progestin components of OCP s (EE and LNG) may critically trigger the metastasis of PTCs. This effect seems to be initiated by upregulating angiogenic factor VEGFA and modulating four EMT-related genes *CDH1*, *CDH2*, *FN1*, and *VIM*. The present results also provided evidence to highlight the possible effects of lncRNAs *MALAT1* and *HOTAIR* in the regulation of the mentioned pathways as well-known epigenetic gene modulators. EE and LNG might exert these effects on PTC cells either through ER-dependent or independent pathways. Taken together, these data are suggestive of the potential role of the exogenous estrogens and progestins found in OCPs in the progression and advancement of PTC tumors.

## Conflict of Interest

There are no conflicts of interest.
